# Acetylated Thioredoxin Reductase 1 Resists Oxidative Inactivation

**DOI:** 10.3389/fchem.2021.747236

**Published:** 2021-09-15

**Authors:** David. E. Wright, Nikolaus Panaseiko, Patrick O’Donoghue

**Affiliations:** ^1^ Departments of Biochemistry, The University of Western Ontario, London, ON, Canada; ^2^ Departments of Chemistry, The University of Western Ontario, London, ON, Canada

**Keywords:** acetylation, enzymology, genetic code expansion, oxidation, post-translational modification, redox biology, selenocysteine

## Abstract

Thioredoxin Reductase 1 (TrxR1) is an enzyme that protects human cells against reactive oxygen species generated during oxidative stress or in response to chemotherapies. Acetylation of TrxR1 is associated with oxidative stress, but the function of TrxR1 acetylation in oxidizing conditions is unknown. Using genetic code expansion, we produced recombinant and site-specifically acetylated variants of TrxR1 that also contain the non-canonical amino acid, selenocysteine, which is essential for TrxR1 activity. We previously showed site-specific acetylation at three different lysine residues increases TrxR1 activity by reducing the levels of linked dimers and low activity TrxR1 tetramers. Here we use enzymological studies to show that acetylated TrxR1 is resistant to both oxidative inactivation and peroxide-induced multimer formation. To compare the effect of programmed acetylation at specific lysine residues to non-specific acetylation, we produced acetylated TrxR1 using aspirin as a model non-enzymatic acetyl donor. Mass spectrometry confirmed aspirin-induced acetylation at multiple lysine residues in TrxR1. In contrast to unmodified TrxR1, the non-specifically acetylated enzyme showed no loss of activity under increasing and strongly oxidating conditions. Our data suggest that both site-specific and general acetylation of TrxR1 regulate the enzyme’s ability to resist oxidative damage.

## Introduction

Human cells actively eliminate reactive oxygen species (ROS) and resolve oxidative damage to proteins using multiple pathways, including the glutathione or thioredoxin (Trx) systems (Schweizer and Fradejas-Villar, 2016). The Trx system includes the selenocysteine-containing protein (selenoprotein) thioredoxin reductase (TrxR1). TrxR1 is a disulfide reductase with specificity for the redox mediator Trx. TrxR reduces a disulfide bond in Trx by catalyzing the oxidation of nicotinamide adenine dinucleotide phosphate (NADPH). The reduced Trx transfers electrons to oxidatively damaged proteins or ROS directly. For example, a pathway that resolves oxidation of methionine residues uses Trx-dependent enzymes to protect the proteome (Kim and Gladyshev, 2007). The resulting oxidized Trx can then be reduced again by TrxR1.

The Trx system is involved in regulating gene expression, embryonic development, cell proliferation, apoptosis, and many other cellular processes (Lu and Holmgren, 2012). In addition to Trx, TrxR1 can also directly reduce other cellular proteins, such as p53, protein disulfide isomerase, glutathione peroxidase, and NK-lysin (Arner et al., 1999; Mustacich and Powis, 2000) as well as low molecular weight ROS, including hydrogen peroxide, lipoic acid, selenite, 15-HPETE, and more (Arner, 2009). The Trx system provides a defense mechanism against ROS generated during oxidative stress, and consequently, alterations in the Trx system are associated with various diseases. TrxR1 is over-active in many aggressive cancers and is an early diagnostic marker (Selenius et al., 2010; Dong et al., 2016). TrxR1 is also an established anti-cancer drug target (Wang et al., 2012), and increased TrxR1 production or activity provides chemotherapeutic resistance to treatments that rely on the production of ROS to kill cells (Roh et al., 2017).

**FIGURE 1:**
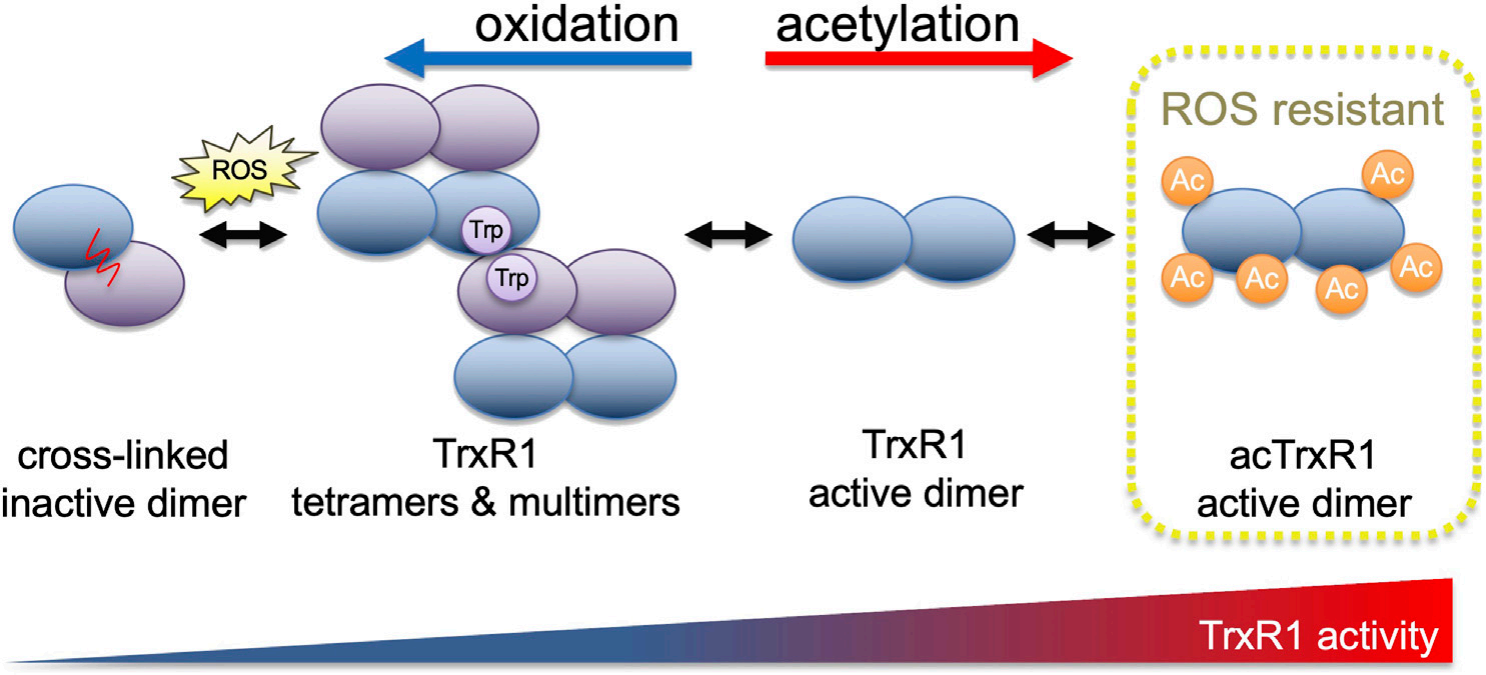
TrxR1 activity is regulated by oxidation and acetylation. With increasing levels of reactive oxygen species, TrxR1 shows increased propensity to form low activity tetramers and higher order multimers (Wright et al., 2018). Oxidation ultimately leads to the formation of a covalent linkage between non-productive TrxR1 monomers, forming inactive cross-linked dimers (Xu et al., 2015). Acetylation of TrxR1, on the other hand, is associated with increased TrxR1 activity, reduced cross-linked dimer formation (Wright et al., 2018), and here we show that acetylated TrxR1 is resistant to oxidation and peroxide-induced multimerization.

TrxR1 exists in an equilibrium of several different quaternary structures (Rengby et al., 2009; Selenius et al., 2010; Xu et al., 2015; Wright et al., 2018). TrxR1 can exist as inactive monomers, or as low active tetramers or higher order oligomers (Rengby et al., 2009; Xu et al., 2015; Wright et al., 2018; Shu et al., 2020). Catalytically competent dimers are the most active form of TrxR1, while inactive cross-linked dimers form because of covalent linkage between opposing subunits in associations between TrxR1 tetramers (Figure 1) (Xu et al., 2015; Wright et al., 2018). In cells, oxidative stress generated by Reactivating p53 and Inducing Tumor Apoptosis (RITA) induces TrxR1 tetramerization and covalent linkage, resulting in reduced activity or inactivation of TrxR1 (Xu et al., 2015). Thus, as oxidative stress increases, one of the cell’s major oxidative stress defense mechanisms is prone to become increasingly ineffective.

Multiple reports document regulation of the Trx system without changes in total protein levels. These studies implicate post-translational modifications as potent regulators of the activity of different Trx system components (Choudhary et al., 2009; Folami Lamoke et al., 2011; Lamoke et al., 2011; Banerjee Mustafi et al., 2014). Three members of the Trx system can be acetylated to increase their activity, including TrxR1 (Wright et al., 2018), Trx1 (Folami Lamoke et al., 2011), and peroxiredoxin (Prx1) (Parmigiani et al., 2008). Acetylation inhibits super-oxidation of Prx1, preventing its oligomerization into higher molecular weight complexes with low peroxidase activity (Parmigiani et al., 2008). In the context of disease, TrxR1 acetylation levels correlated positively with the level of oxidized cellular proteins in a mouse model of cardiomyopathy (Banerjee Mustafi et al., 2014). The oxidative stress generating anti-cancer compound RITA reduces TrxR1 activity in cell cultures by altering the oligomerization status of TrxR1 and increasing levels of inactive and cross-linked dimers (Xu et al., 2015). These reports suggest a relationship between acetylation, oligomerization, and oxidative damage in components of the Trx system. Although TrxR1 is known to be acetylated in response to oxidative stress (Banerjee Mustafi et al., 2014), the function of TrxR1 acetylation under oxidizing conditions is unknown.

Proteomic studies in Jurkat T lymphocytes, A549 cells and related non-small cell lung cancer cell lines have identified acetylation of TrxR1 at five distinct sites (Choudhary et al., 2009; Hornbeck et al., 2015; Wu et al., 2015). We showed that single acetylation at three of these sites in TrxR1 resulted in a 1.5 to 3-fold increase in enzyme activity (Wright et al., 2018). Because acetylated TrxR1 shows increased activity, we hypothesized that the acetylation of TrxR1 may serve as a mechanism to maintain TrxR1 activity under oxidizing conditions associated with increased ROS levels. To test this hypothesis, we used protein biochemistry to precisely measure the activity of acetylated TrxR1 variants over a broad range of peroxide concentrations that models the relevant range of ROS levels encountered by cells. We also generated a non-specifically acetylated TrxR1 using aspirin as a model acetyl donor. Together our findings suggest that acetylation is a potent mechanism to regulate TrxR1 activity that also allows the enzyme to evade oxidative inactivation.

## Materials and Methods

### Plasmids and Strains

**FIGURE 2:**
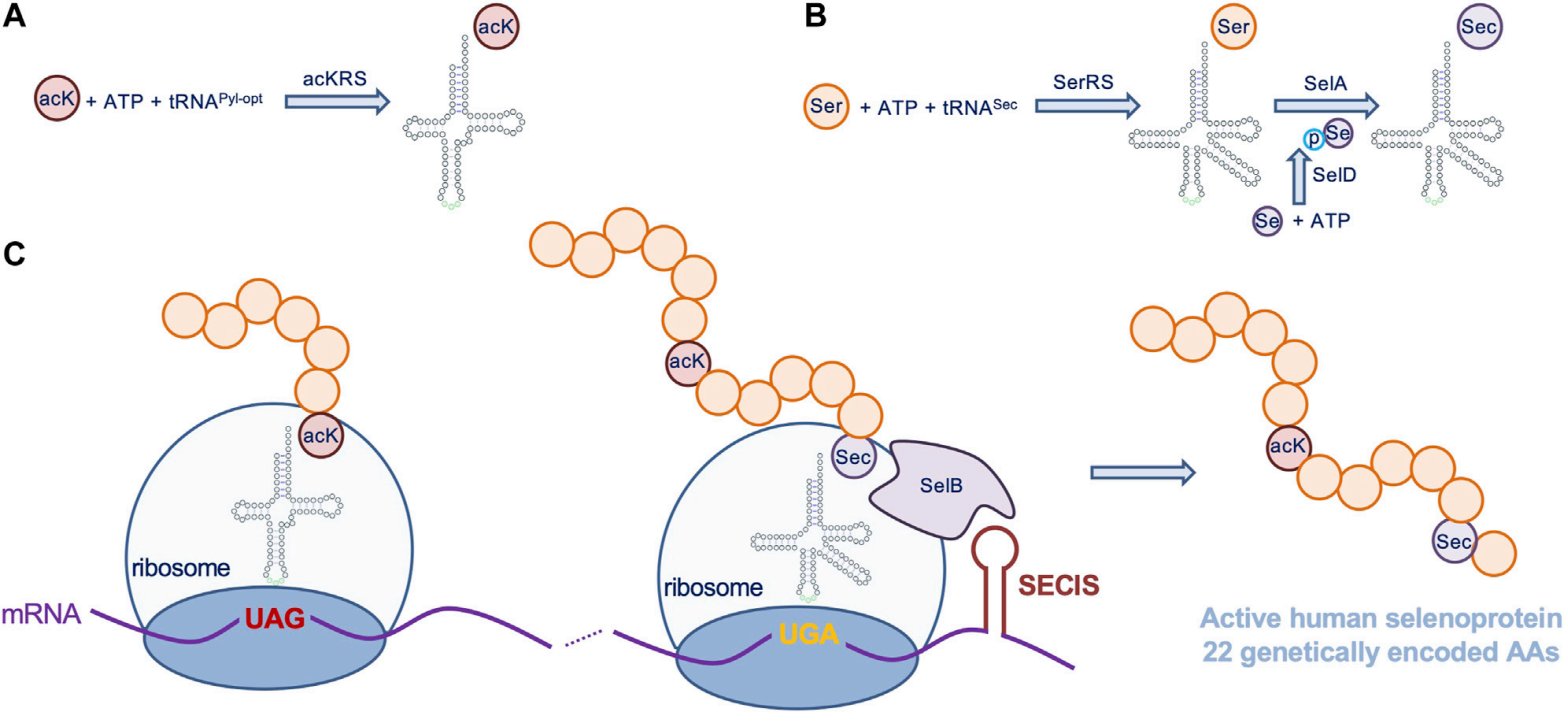
Genetic code expansion to incorporate acK and Sec in TrxR1. Expressed from a plasmid in *E. coli*, **(A)** AcKRS ligates the UAG-decoding tRNA^Pyl-opt^ with acK, **(C)** allowing insertion of acK at UAG codons. **(B)** In the endogenous Sec incorporation pathway (reviewed in (Serrao et al., 2018)), *E. coli* SerRS ligates tRNA^Sec^ with Ser, selenophosphate synthetase (SelD) produces selenophosphate (pSe), and selenocysteine synthase (SelA) uses the products of these reactions to convert Ser-tRNA^Sec^ to Sec-tRNA^Sec^. An endogenous *E. coli* elongation factor (SelB) recruits Sec-tRNA^Sec^ to a UGA codon by also binding to a selenocysteine insertion sequence (SECIS) included in the 3′ untranslated region of the TrxR1 mRNA, allowing Sec incorporation at a specific UGA codon (C). Together the acK and Sec systems can be used to produce active human TrxR1 protein with 22 genetically encoded amino acids.

The plasmid pET-pylT-TrxR1 contains a His-tagged Human TrxR1 (isoform 4) with an in-frame UGA codon (Sec551) followed by the *E. coli* selenocysteine insertion sequence (SECIS) RNA-hairpin loop (derived from the *E. coli* FdhF gene) in the 3′ untranslated region (3’ UTR), which directs Sec-insertion at the UGA551 codon in recombinantly produced human TrxR1 variants from *E. coli* (Figure 2) (Wright et al., 2018). UAG stop codons inserted at positions 141, 200, or 307 in the TrxR1 gene allows for site-specific insertion of *N*_ε_-acetyl-L-lysine (AcK) when co-expressed with plasmids bearing a pyrrolysyl-tRNA synthetase mutant specific for Ack (AckRS) Guo et al. (2014) and an optimized (Fan et al., 2015) UAG-decoding tRNA^Pyl^ (pTech-acKRS-tRNA^Pyl-opt^) (Figure 2); (Wright et al., 2018).

### TrxR1 and acTrxR1 Protein Purification

*E. coli* BL21 (DE3) (Invitrogen) was co-transformed with pET and pTech vectors. Preparative cultures (1 l) for each transformed strain were incubated with shaking at 37°C in lysogeny broth (LB) supplemented with 5 mM AcK (A4021-5G Sigma), 10 µM sodium selenite (10102-18-8, AlfaAesar), and appropriate antibiotics (100 μg/ ml ampicillin (BP1760-25, Fisher) for pET and 34 μg/ ml chloramphenicol (02930-100G, Ampresco) for pTech). We employed a previously optimized protocol for production of the selenoproteins in *E. coli* (Wright et al., 2018). At A_600_ = 1.2, the temperature was reduced to 20°C. At A_600_ = 1.5, 1 mM isopropyl β-d-1-thiogalactopyranoside (IPTG) (BP1755-10, Fisher) was added and the cells then produced protein for a further 16–24 h. Cells were harvested by centrifugation and stored at −80°C until further use. TrxR1 variants were purified as previously (Wright et al., 2018). Briefly, cell pellets were resuspended in 30 ml phosphate buffer (100 mM potassium phosphate (PB0445, Biobasic), pH 7.2, 10% glycerol (CA97063-892, VWR)) supplemented with lysozyme (1 mg/ ml) (12650-88-3, Biobasic) and disrupted by sonication. Following centrifugation at 6,250 × *g*, cell lysate was purified by affinity chromatography using Ni^2+^-Nitrilotriacetic acid (NTA) resin (HisPur™ Ni-NTA Resin, PI88222, Fisher), as previously (Wright et al., 2018). Purified TrxR1 variants were stored in 100 mM potassium phosphate, pH 7.2, 50% glycerol at −80°C until use.

### TrxR1 Activity Assays

TrxR1 activity was assessed using 5,5-dithio-bis-(2-nitrobenzoic acid) (DTNB) (D8130-5G, Sigma) also known as Ellman’s reagent to detect the rate and level of reductive activity from TrxR1. The colorimetric reaction is followed by measuring reduction of DTNB to 2-nitro-5-thiobenzoate (TNB), which absorbs at 412 nm (A_412_). Each reaction contains 250 nM TrxR1, 300 µM Nicotinamide adenine dinucleotide phosphate (NADPH) (N5130-25 MG, Sigma) and 5 mM DTNB in buffer containing 100 mM potassium phosphate, 1 mM Ethylenediaminetetraacetic acid (EDTA) (E4378-25G, Sigma), pH 7.0. Reactions were started by the addition of DTNB to a solution containing TrxR1 and NADPH, for a final volume of 100 µl in a 96 well plate. Measurements were taken in a Biotek Synergy H1 microplate reader every 1 min over a 1-h time course. All assays were performed using three independent enzyme reactions for each condition tested.

### Peroxide and Aspirin Incubations

TrxR1 variants were also incubated with increasing concentrations of peroxide and then assessed as above for activity. Each TrxR1 and acTrxR1 enzyme variant (1 µM) was incubated in phosphate buffer (100 mM potassium phosphate, 1 mM EDTA, pH 7.0) with increasing peroxide concentrations (0–500 µM H_2_O_2_ (16,911-250ML-F, Sigma)) for 1 h in a total volume of 200 μl at 37°C. To generate non-site-specifically acetylated TrxR1, 500 nM TrxR1 was incubated with increasing concentrations (0–15 mM) of aspirin (A5376-250G, Sigma) in phosphate buffer for 1 h at 37°C. Activity assays with aspirin-treated and untreated TrxR1 were conducted exactly as above.

### Western Blotting

Purified protein samples were suspended in 1 × sodium dodecyl sulfate (SDS) loading buffer (250 mM Tris-HCl pH 6.8, 40% glycerol (v/v), 10% SDS (w/v) (SB0485, Biobasic), 0.05% bromophenol blue (w/v) (0449-25G, Amresco), 5% 2-mercaptoethanol (M6250-100ML, Sigma)) and heated for 5 min at 95°C. Samples were then loaded in 15% SDS-polyacrylamide gels and electrophoresed. Following this, a PVDF membrane was soaked in methanol for 1 min. Both the SDS gel and membrane were soaked in transfer buffer (0.025 M Tris-HCl (TRS001.5, Bioshop Canada), pH 9.5, 0.192 M Glycine (56-40-6, Fisher), 20% (v/v) Methanol (CA71007-742, VWR), 0.5% (w/v) SDS) for 15 min. The blot was carried out with a TransBlot Turbo Transfer System (BioRad) at 15 V with 1.3 A for 15 min. The membrane was incubated in blocking solution (5% (w/v) skim milk powder (LP0031, Oxoid), 0.1% (v/v) Tween20 (9,005–64–5, Ampresco), 1x PBS (137 mM NaCl (BP358-212, Fisher), 0.027 mM KCl (7447-40-7, Anachemia), 10 mM Na_2_HPO_4_ (SDB0487, Biobasic), and 2 mM KH_2_PO_4FF_) for 1 h, shaking, at room temperature. Then the membrane was incubated overnight with the primary antibody (anti-acetyl Lysine antibody from rabbit, Abcam ab80178; or anti-TrxR1 antibody, Santa Cruz Biotechnology sc28321) at 1:1,000 in blocking solution at 4°C overnight. Three 10-min washes with wash solution (0.5% (w/v) skim milk powder, 0.1% Tween20, 1x PBS) were conducted, shaking, at room temperature, followed by a 2 h incubation with a secondary antibody (Rabbit IgG HRP Linked F (ab′)2, GENA9340; Sigma) at 1:5,000 in wash solution at room temperature shaking. Next, three 10-min washes with PBS-tween (0.1% (v/v) Tween20, 1x PBS) were conducted, followed by a final wash with 1x PBS, shaking at room temperature. Clarity Western ECL Substrate (1,705,061; Biorad) was used for signal detection and chemiluminescent imaging was performed on a Chemidoc XRS + (Biorad).

### Mass Spectrometry

For the MS/MS analysis of the aspirin acetylated and untreated TrxR1, gels were loaded with 0.31 ug of purified TrxR1 protein. Following SDS-PAGE, a 1 mm circular slice was picked from the gel using an Ettan Robotic Spot-Picker and submitted for proteolytic digestion (Trypsin) and peptide extraction at the Functional Proteomics Facility at the University of Western Ontario. Liquid chromatography and tandem mass spectrometry (LC-MS/MS) analyses of TrxR1 and aspirin-acetylated TrxR1 were performed at the Biological Mass Spectrometry Laboratory at The University of Western Ontario. Gel slices were de-stained with 50 mM ammonium bicarbonate (09830, Sigma) and 50% acetonitrile (00687, Sigma). The protein samples were reduced with 10 mM dithiothreitol (BP25641, Fisher), alkylated with 55 mM acrylamide (BP1406-1, Fisher), and digested with 5 ng/ μl trypsin (Promega). LC-MS/MS was performed using a Q-Tof Micro mass spectrometer (Waters) equipped with a Z-spray source in positive ion mode (+ 0.1% formic acid) or using Thermo Scientific LTQ-Orbitrap XL mass spectrometer. The data were analyzed and visualized using PEAKS software (Bioinformatic Solutions, Inc, Waterloo, Ontario).

### Statistical Analysis

All activity assays were conducted in at least three independent enzyme reactions, including enzymes from independent preparations. A no enzyme (-TrxR1) control was subtracted from all reactions. All error bars represent one standard deviation, and *p*-values were calculated from a one-way analysis of variance.

## Results

### Purification of AcK and Selenocysteine-Containing TrxR1 Variants

We used genetic code expansion to produce TrxR1 variants in *E. coli* with 22 different genetically encoded amino acids, including the non-canonical amino acids (ncAAs) acetyl-lysine (AcK) and selenocysteine (Sec) (Figure 2). We previously described purification, biochemical characterization and mass spectrometry analysis of each of TrxR1 variants to determine activity and verify incorporation of AcK at K141, K200, or K307 as well as Sec incorporation at the key active reside Sec551 (Wright et al., 2018). Briefly, we used a genetic code expansion system based on a PylRS mutant with activity for ligating AcK to tRNA^Pyl^, which decodes amber (UAG) stop codons. Thus, by placing a UAG codon at the positions 141, 200 or 307 in our expression construct (Figure 2), we generated site-specifically acetylated TrxR1 variants.

Our TrxR1 expression construct also has a *E. coli* SECIS appended to the 3-untranslated region of our recombinant human TrxR1 gene. The SECIS element recruits the endogenous Sec-incorporation machinery to recode a UGA codon at position 551 from stop to Sec (Figure 2). Bacterial cultures must be supplement with sodium selenite to enable Sec formation (see Materials and Methods). The data generated below are based on multiple independent enzyme preparations, of which representative purified samples were visualized by SDS-PAGE (Supplementary Figure S1).

### Resistance of Site-specifically Acetylated TrxR1 to Oxidative Damage

Because our work showed increased activity of site-specifically acetylated TrxR1 Wright et al. (2018) and previous studies implicated increased TrxR1 acetylation as a response to oxidative damage (Banerjee Mustafi et al., 2014), we hypothesized that acTrxR1 may be more active under oxidizing conditions or even resistant to oxidative damage. In cells, there are many sources of ROS, including superoxide, hydroxy, and nitric oxide radicals as well as H_2_O_2_, which is also an important signaling molecule (Bae et al., 2011).

**FIGURE 3:**
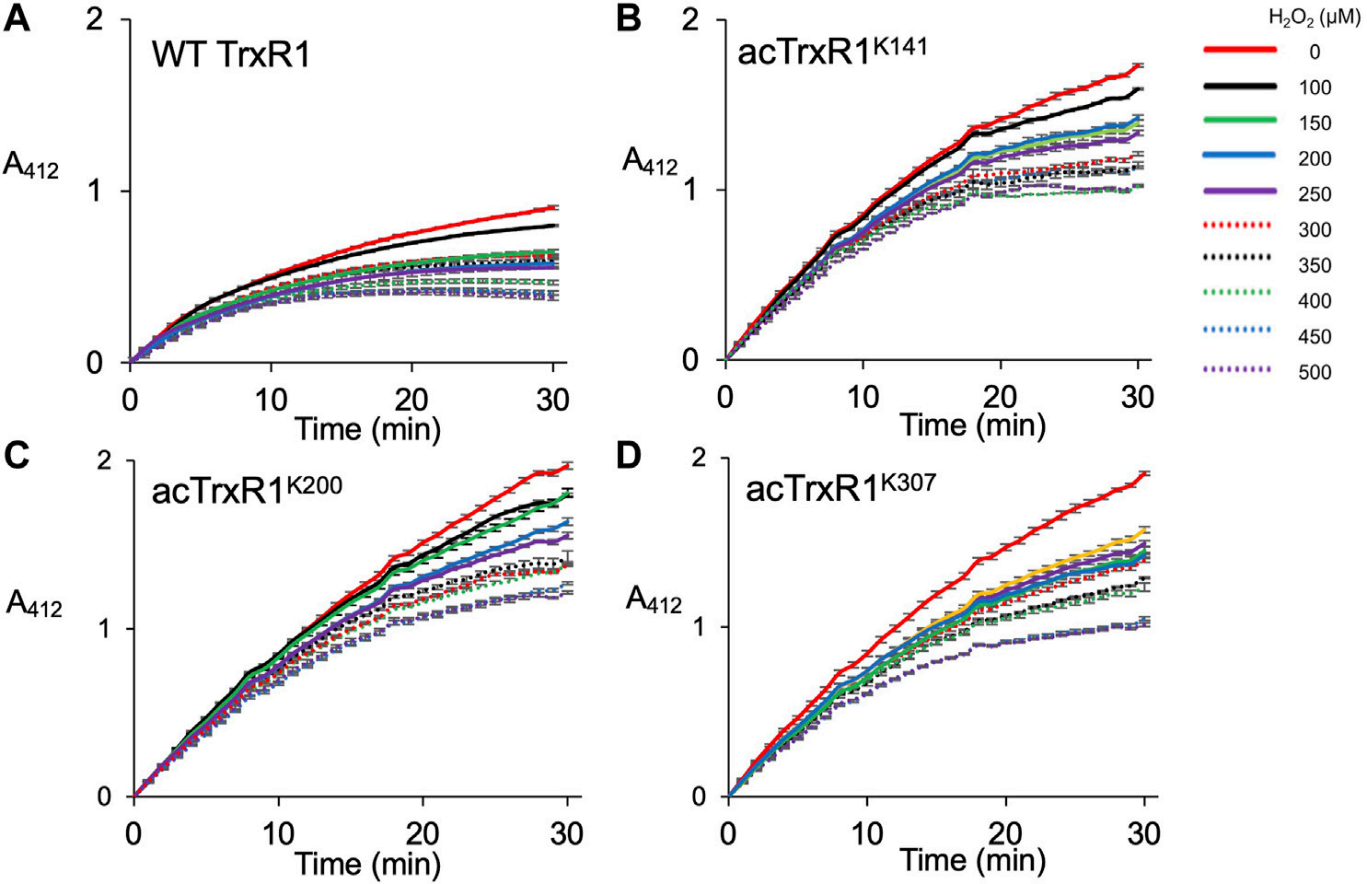
Activity of un-modified and acTrxR1 variants with increasing peroxide levels. Purified TrxR1 variants were incubated with varying concentrations of buffer or H_2_O_2_ ranging from 0 to 500 µM for 1 h at 37°C. Following incubation, enzyme activity with the TrxR1 substrate DTNB was determined by following absorbance at 412 nm (A_412_). Activity was measured for **(A)** wild-type TrxR1 and site-specifically acetylated variants **(B)** acTrxR1^K141^, **(C)** acTrxR1^K200^, and **(D)** acTrxR1^K307^. Error bars represent ± 1 standard deviation about the mean of three independent enzyme reactions.

**FIGURE 4:**
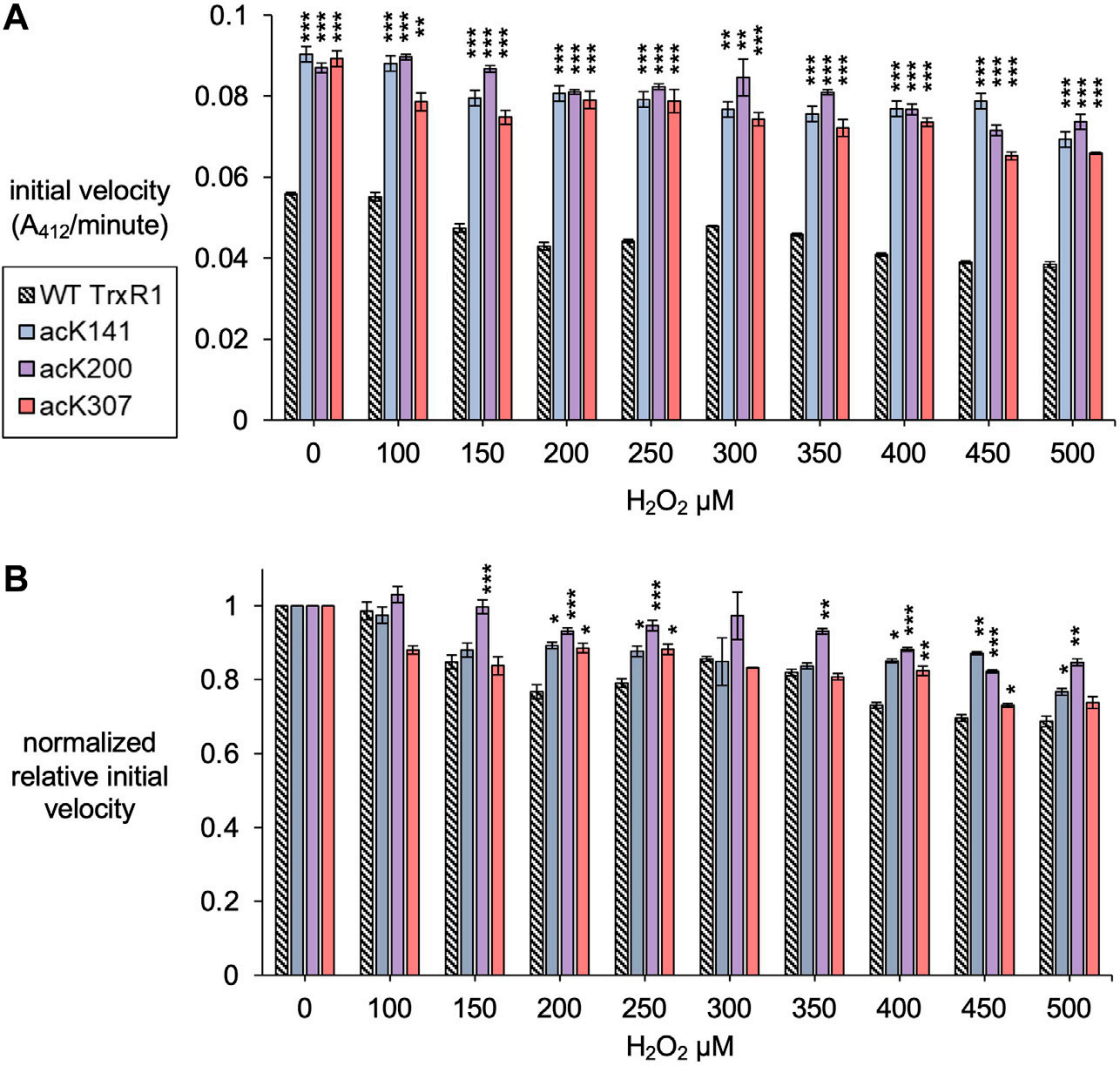
Initial velocity of un-modified and specifically acetylated TrxR1 variants with increasing oxidative damage. **(A)** Initial velocity of each TrxR1 variants under the indicated H_2_O_2_ concentrations was calculated from the kinetic data (Figure 3). **(B)** To show relative changes in activity, the initial velocities were normalized by the activity of each variant under normal conditions (0 µM H_2_O_2_). Error bars represent ± 1 standard deviation about the mean of three independent enzyme reactions. Significant differences are annotated (*p < 0.05; **p < 0.01; ***p < 0.001) to compare acTrxR1 variants to WT TrxR1 in **(A)** or to compare the activity at each peroxide concentration to the 0 µM peroxide condition in (B).

In mammalian cells, physiological concentrations of H_2_O_2_ usually range from 0.1 to 10 μM, while cell stress responses are associated with greater peroxide concentrations in range of 10–500 µM (Sies, 2017). In apoptotic or necrotic human melanoma cells, peroxide levels can rise to more than 500 µM (Clement et al., 1998). To mimic oxidative damage that occurs in cells, we designed a series of experiments to measure the activity and initial velocity of purified TrxR1 and acTrxR1 variants under a range of peroxide concentrations (0–500 µM) encountered by normal as well as stressed cells. We first measured the catalytic activity of site-specifically acetylated TrxR1 (acTrxR1) variants as well as the wild-type TrxR1 (Figure 3). As previously (Wright et al., 2018), we found that under normal conditions (0 µM H_2_O_2_), each of the acTrxR1 variants showed substantially more activity (∼1.5-fold) than the un-modified TrxR1 (Figure 3). Increased activity of acTrxR1 variants is evident in both the maximal level of DTNB reduced (Figure 3) as well as the initial velocity observed during the reaction time course (Figure 4).

All TrxR1 variants showed reduced catalytic activity with increasing concentrations of hydrogen peroxide (H_2_O_2_). At all concentrations of H_2_O_2_, we observed significantly more absolute activity of each acTrxR1 variant compared to wild type (Figure 4A). Even at the highest peroxide concentrations, the absolute maximal activity level of acTrxR1 variants was still higher than that we observed for un-modified TrxR1 even without peroxide treatment (Figure 3). The data suggest that the acTrxR1 variants are resistant to oxidation.

Based on the initial velocity observed in the DTNB reduction reactions, we then calculated the absolute (Figure 4A) and relative (Figure 4B) reduction in catalytic rate observed in wild type compared to acTrxR1 variants with increasing peroxide concentration. The absolute reaction rates responded to oxidation similarly as noted above. Namely, the acTrxR1s showed reduced initial velocities with increasing peroxide that was always significantly greater than the observed for the wild type TrxR1 (Figure 4A). The acTrxR1 variants also show robust or partial resistance to oxidation even when the maximal catalytic rates for TrxR1 and acTrxR1 are both normalized to 1.0. After normalization to the un-modified TrxR1, both acTrxR1^K141^ and acTrxR1^K200^ showed significantly less relative activity reduction at each peroxide concentration tested (Figure 4B). The data demonstrate that acTrxR1^K141^ and acTrxR1^K200^ are significantly more resistant to oxidation than the un-modified enzyme. For the acTrxR1^K307^ variant, we found similar resistance to oxidation in the normalized relative activity rates at about half of the peroxide concentrations tested from 200–450 µM H_2_O_2_. The data indicate that site-specific acetylation of TrxR1 is protective against the effects of oxidative damage.

We also monitored the TrxR1 proteins in the above reactions using SDS-PAGE. We visualized the TrxR1 variants under each condition using Coomassie staining and Western blotting with an anti-TrxR1 antibody (Supplementary Figure S2). At the highest peroxide concentration, we observe some degradation of the TrxR1 protein. The western blot revealed the presence of covalently linked high molecular weight TrxR1 oligomers following oxidation with H_2_O_2_ (Supplementary Figures S2, S3). For unmodified TrxR1, the high molecular weight complexes are visible already at 100 µM peroxide (Supplementary Figure S2A). Each of the acTrxR1 variants showed less accumulation of the TrxR1 multimers at each peroxide concentration. For the acTrxR1 variants, we observed a similar level of oligomerization to un-modified TrxR1 only at 500 µM peroxide (Supplementary Figures S2B–S2D). Quantification of the western blots confirmed that all acTrxR1 variants had a statistically significant reduction in the accumulation of higher molecular weight TrxR1 complexes compared to un-modified TrxR1 at all peroxide concentrations tested for acTrxR1^K307^, from 100 to 400 µM H_2_O_2_ for acTrxR1^K200^, and from 200 to 400 µM H_2_O_2_ for acTrxR1^K141^ (Supplementary Figure S3).

### Aspirin Acetylates TrxR1 and Provides Robust Resistance to Oxidative Damage

Protein acetylation can occur specifically in cells resulting from the activity of acetyltransferases, but also non-specifically through interactions with acetyl donors including acetyl-CoA or certain drugs, such as aspirin (Tatham et al., 2017). We hypothesized that like specific or programmed acetylation of TrxR1, general or non-specific acetylation of the enzyme may also provide resistance to oxidative damage.

**FIGURE 5:**
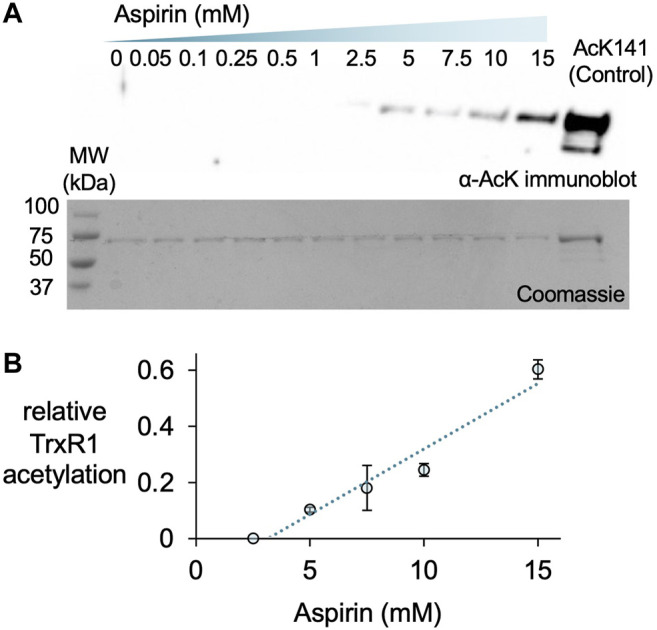
Aspirin acetylates TrxR1. TrxR1 was incubated with varying concentrations of aspirin (0–15 mM) for 1 h at 37°C. Following incubation, 0.31 µg of protein samples were separated on a 15% SDS-PAGE and visualized **(A)** after immunoblotting with an anti-acetyl-lysine antibody (α-AcK) or staining with Coomassie blue dye. The acTrxR1^K141^ produced with genetic code expansion served as a positive control. Based on these data, we calculated the relative level of TrxR1 acetylation compared to the acTrxR1^K141^ control **(B)**, which increased linearly with increasing aspirin concentration.

A previous report used incubated HeLa cells with a range of aspirin concentrations from 0.5 to 20 mM to generate aspirin-mediated and non-specific acetylation of many cellular proteins, including TrxR1 (Tatham et al., 2017). Thus, we used western blotting to detect acetylation of purified TrxR1 resulting from incubation with aspirin over a similar concentration range (Figure 5). To estimate the level of acetylated TrxR1, we used our site-specifically modified acTrxR1^K141^ as control. The untreated and un-modified TrxR1 showed no reactivity with an anti-acK antibody, while robust detection was evident with the acetylated control sample (acTrxR1^K141^). Lower concentrations of aspirin did not lead to detectable acetylation, but at higher concentrations significant acTrxR1 is clearly present in the blot (Figure 5A). We then used the Coomassie stained gel to normalize the amount of protein loaded and determined the relative intensity of bands in the anti-acK immunoblot. We showed previously that acTrxR1^K141^ has stoichiometric incorporation of acK at the 141 site (Wright et al., 2018). Thus, based on comparison to the control, the relative level of acetylation following aspirin incubation reached a level ∼65% of that observed in acTrxR1^K141^ (Figure 5B).

**FIGURE 6:**
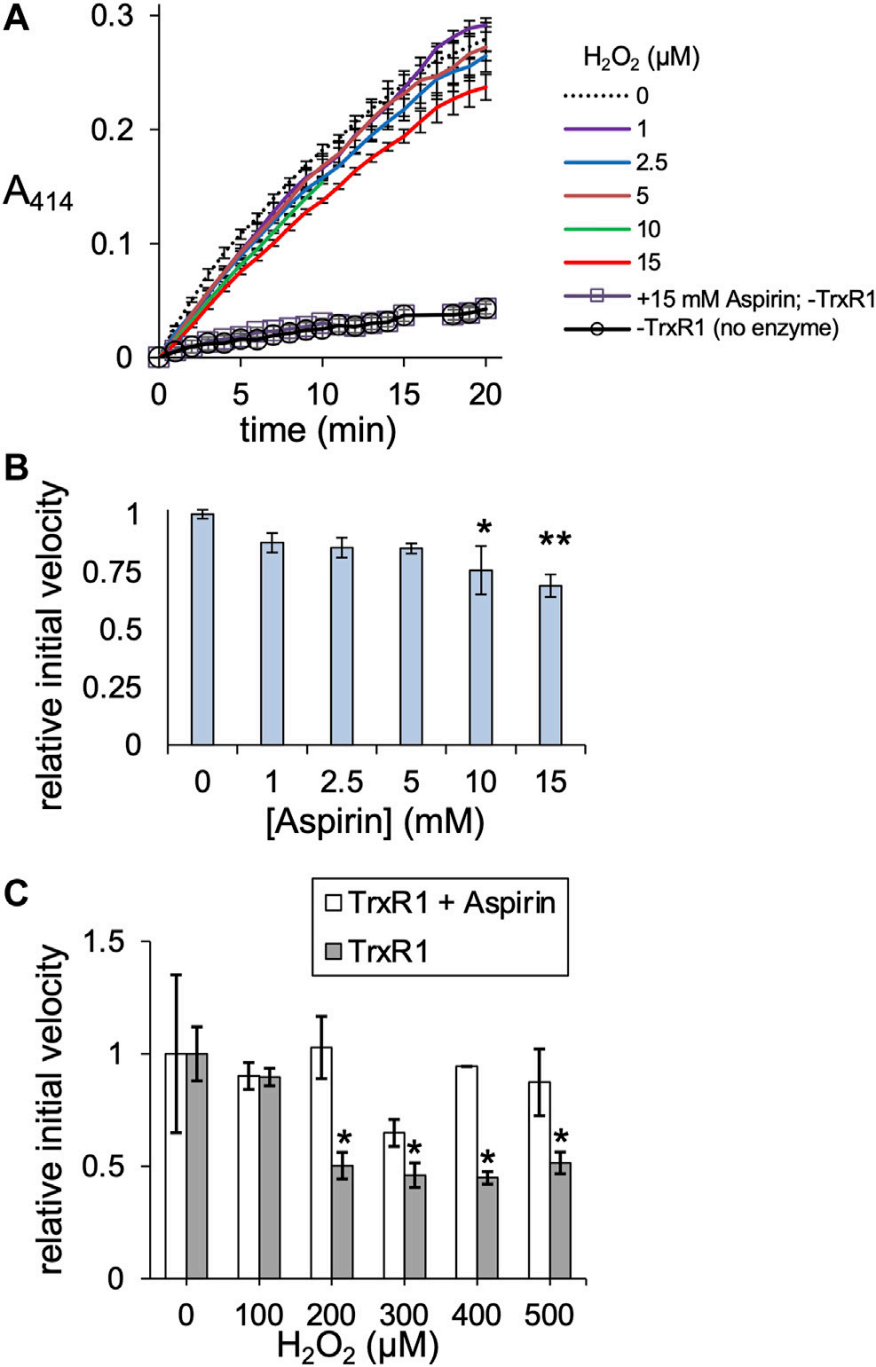
TrxR1 activity following aspirin incubation. **(A)** TrxR1 variants were incubated with 0–15 mM aspirin for 1 h at 37°C, followed by TrxR1 activity assays using DTNB. Negative controls containing no TrxR1 (-TrxR1) with 0 or 15 mM aspirin was also incubated for 1 h at 37°C, followed by the DTNB assay. **(B)** Initial velocity was calculated for TrxR1 activity based on the data in **(A)**. Statistical analysis comparing the activity at each aspirin concentration **(B)** to the untreated enzyme showed significant reductions in activity only at 10 and 15 mM aspirin (*p < 0.05; **p < 0.005). **(C)** TrxR1 was incubated with 15 mM aspirin for 1 h at 37°C, followed by incubation with 0–500 µM H_2_O_2_ for 1 h at 37°C. Following incubations, the initial velocity of TrxR1 activity was determined from TrxR1 activity assays and normalized to the initial velocity with 0 µM H_2_O_2_. Statistical analysis comparing the activity at each peroxide concentration show significant reductions in activity in the un-modified TrxR1 only and not in the aspirin treated enzyme (*p < 0.05). Error bars represent ± 1 standard deviation about the mean of three independent enzyme reactions.

We next compared the activity of TrxR1 and non-specifically acetylated TrxR1 following incubation with aspirin. In contrast to site-specifically acTrxR1 variants, aspirin incubation results in a slight decrease to ∼70% of wild-type TrxR1 activity that was significantly lower only at the highest aspirin concentrations (>5 mM). Controls lacking enzyme (-TrxR1) and with or without aspirin showed no ability to catalytically reduce DTNB (Figure 6A). To test the ability of non-specifically acetylated TrxR1 to resist oxidative damage, unmodified TrxR1 was incubated with or without aspirin, followed by a second incubation of 1 h with increasing peroxide concentrations (0–500 µM H_2_O_2_) before DTNB reduction activity assays were conducted. In contrast to un-treated TrxR1, TrxR1 incubated with aspirin showed no statistically significant decrease in relative activity in response to any of the H_2_O_2_ concentrations tested (Figure 6C). The data suggest that aspirin mediated TrxR1 acetylation provides robust resistant to oxidative damage.

### Location of TrxR1 Acetylation Sites Following Aspirin Treatment

We used tandem mass spectrometry to identify acetylation sites in purified wild-type TrxR1 following incubation with or without aspirin. Following incubation, the purified protein samples were digested with trypsin and analyzed by LC-MS/MS. We searched the spectra for the possibility of multiple modifications, including lysine acetylation. In the un-treated sample, we identified acetylation only at K307 according to a single peptide hit (Supplementary Table S1), perhaps due to acetylation by acetyl-phosphate in *E. coli* (Weinert et al., 2013). Based on our observations in the acK immunoblot (Figure 5), we can conclude that the untreated TrxR1 has low levels of acetylation at this site.

**FIGURE 7:**
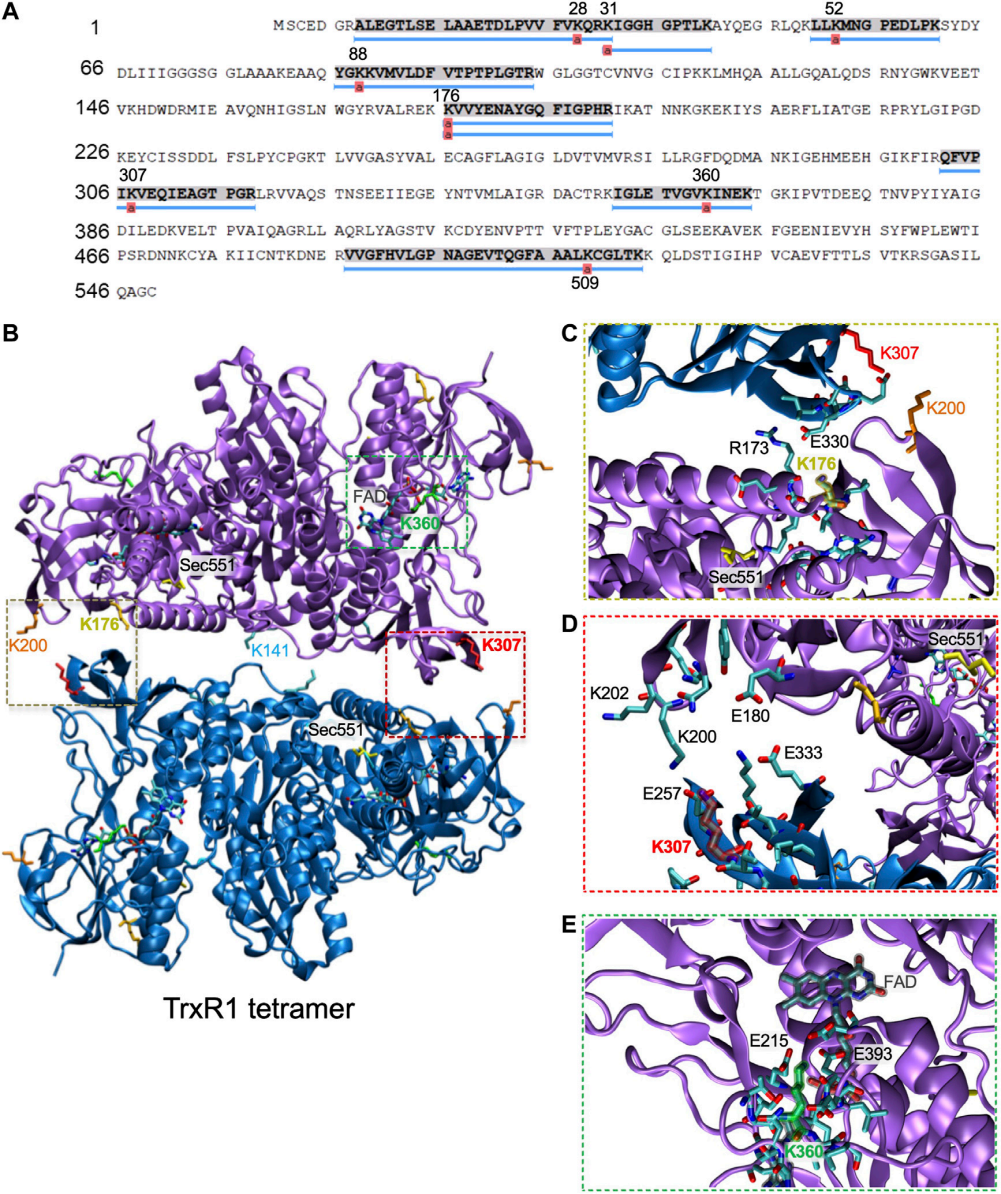
Mapping acetylation sites in TrxR1 to the quaternary structure. **(A)** Coverage map showing acetylation sites identified by LC-MS/MS following incubation of TrxR1 with the non-specific acetyl donor aspirin. **(B)** Lysine acetylation sites were mapped onto the TrxR1 tetramer structure (PDB codes 4KPR and 3EAN (Xu et al., 2015)): K176 (gold), K307 (red), K360 (green), and K307 (red). Sec551 (yellow) was modeled based on data from the structure 3ean (Rengby et al., 2009). The FAD co-factor is highlighted (grey). Close-up views show interactions with **(C)** K176, **(D)**, K307 **(E)**, and K360. Structures were drawn using VMD (Humphrey et al., 1996).

In contrast, mass spectrometry on aspirin treated TrxR1 identified many new and different lysine acetylation sites supported with multiple, high quality peptides hits to the spectra (Figure 7; Supplementary Figure S4; Supplementary Table S1). These data provide strong evidence that aspirin incubation with TrxR1 leads to acetylation at K28, K31, K52, K88, K176, K307, K351, and K360 (Figures 7A; Supplementary Figures S4; Supplementary Table S1).

## Discussion

### Interplay Between Acetylation and Oxidation in the Trx System

Many proteins are acetylated or hyper-acetylated in conditions of oxidative stress characterized by elevated levels of ROS (Santo-Domingo et al., 2020). Increased acetylation of lysine residues is a common response to oxidative stress documented in diverse proteins, including histones (Gu et al., 2013), FoxO Daitoku et al. (2011) and zinc-finger Wu et al. (2018) transcription factors, tRNA synthetases (Cao et al., 2017), and superoxide dismutase (Ozden et al., 2011). The acetylation status of the Trx system in conditions of oxidative stress is not yet completely characterized; however, increased acetylation of TrxR1 has been linked to oxidative stress in a mouse model of cardiomyopathy (Banerjee Mustafi et al., 2014).

Different components of the Trx system, including TrxR1, Trx, and Prx are acetylated at multiple lysine residues in mammalian cells (Choudhary et al., 2009; Hornbeck et al., 2015; Wu et al., 2015). We and others have found that acetylation increases the activity of TrxR1 (Wright et al., 2018), Trx1 (Folami Lamoke et al., 2011), and Prx1 (Parmigiani et al., 2008). Data from these studies suggest an emerging theme for how acetylation regulates the activity of the Trx system. Our work documented the ability of site-specific acetylation to increase TrxR1 activity by reducing oligomerization (Wright et al., 2018), and here we found acetylation also provided robust resistance to oxidative damage and peroxide-induced TrxR1 oligomer formation.

In studies of Trx, TrxR1, and Prx (Parmigiani et al., 2008), oxidation leads to the formation of low activity oligomers (Parmigiani et al., 2008; Xu et al., 2015), while acetylation provide resistance to oxidative damage and oligomerization (Parmigiani et al., 2008; Wright et al., 2018). Trx acetylation was linked to increased Trx activity in post-mortem diabetic retinas (Folami Lamoke et al., 2011). Much like Prx and TrxR1, Trx oligomerization is induced by oxidation, whereby Trx forms inactive dimers (Ren et al., 1993). The potential for Trx acetylation to counteract oxidation is not yet known. Acetylation of Prx prevents overoxidation and oligomerization (Parmigiani et al., 2008), while TrxR1 acetylation also provides resistance to oxidative inactivation Figures 4, 5 and prevents oligomerization (Supplementary Figures S2, S3); (Wright et al., 2018). Thus, acetylation may play a similar for Trx, which is an interesting area of future study.

The peroxiredoxins, Prx1 and Prx2, are both acetylated by histone acetyltransferase (HAT) *in vitro*, leading to increased activity and resistance to oxidation (Parmigiani et al., 2008). Human prostate cancer cells (LAPC4) readily acetylate Prx1 as Lys197, and these cells as well as another prostate line (LNCaP4) exposed to 25–100 µM H_2_O_2_ produced increasing amounts of high molecular mass Prx oligomers (Parmigiani et al., 2008), which are associated with reduced Prx peroxidase activity (Moon et al., 2005). In order to measure the ability of the acetylated enzyme to resist oxidative stress, the activity of acetylated recombinant Prx1 was measured in up to 2 mM H_2_O_2_. In the presence of 100 μM H_2_0_2_, a 20% increase in Prx1 activity was associated with acK197 compared to the unmodified enzyme. Compared to our measurements with TrxR1 at the same peroxide concentration, we found 60–70% increased rate of activity in the acTrxR1 variants compared to the unmodified enzyme.

Here, we presented novel biochemical evidence that acetylation prevents TrxR1 activity loss in response to oxidative damage. Similarly to findings regarding Prx (Parmigiani et al., 2008), our studies suggest that site-specific acetylation increases TrxR1 activity Wright et al. (2018) and regulates TrxR1 oligomerization to prevent oxidative inactivation due to covalently linked multimer formation. TrxR1 forms these inactive cross-linked dimers due to oxidation at Trp114 in the dimer-dimer interface (Xu et al., 2015). Taken together, these studies highlight acetylation as a potent regulatory mechanism for Trx system activity that may provide protection against oxidative damage in cells at a time when the Trx system is most in need. The crosstalk between acetylation and oxidation is an open area of research for future work in the Trx system and beyond.

### Non-specific Acetylation of Proteins in the Context of Oxidative Stress

We also demonstrated acetylation by the non-specific acetyl-donor aspirin provides TrxR1 with resistance to oxidative damage. We did not detect non-specific acetylation at the K141 or K200 sites, indicating these sites may not be accessible to aspirin. We previously found that acK307 reduces the formation of low activity tetramers and cross-linked inactive dimers (Wright et al., 2018). K176 is also in the dimer-dimer interface and participates in interactions with E300 of the opposing subunit (Figure 7C). K307 is localized close to K176 and in the dimer-dimer interface (Figure 7D). Both K176 and K307 participate in salt bridge interactions with the opposing subunit, and acetylation of these sites will weaken or eliminate key interactions that stabilize the TrxR1 tetramers. Other aspirin-mediated acetylation sites, K351 and K360, are located near the FAD cofactor binding site close to the active site of TrxR1 (Figure 7). Acetylation of K351 may disrupt interactions with the FAD co-factor, while acetylation of K360 may disrupt salt bridge interactions between K360 and E215 and E393, which are in the vicinity of the FAD co-factor (Figure 7E). Because the position and environment of FAD is critical for TrxR1 function, acetylation at K351 and K360 may be responsible for the marginal loss of TrxR1 activity we observed at high aspirin concentrations (Figure 6B).

Various metabolic compounds can non-enzymatically acetylate proteins, including acetyl-CoA (Weinert et al., 2014). Mitochondrial proteins show increased non-enzymatic acetylation by acetyl-CoA produced alongside energy metabolism (Wagner and Payne, 2013). In addition to metabolites, certain drugs, such as aspirin, can lead to acetylation of many proteins (Tatham et al., 2017). Indeed, the ability of aspirin to reduce inflammation and relieve pain is due to acetylation of the cyclooxygenases COX-1 and COX-2 in their active sites (Loll et al., 1995). Studies in mammalian cells demonstrated aspirin-mediated acetylation of p53 and many other cellular proteins using radiolabeled aspirin and immunodetection (Alfonso et al., 2009). These studies provide a direct link between non-enzymatic acetylation of proteins under conditions that generate ROS or in response to certain medications. Non-enzymatic acetylation may play an important role in TrxR1 and other proteins that maintain the balance between oxidative damage and antioxidant defense.

### Relevance of Trx System Acetylation to Disease

Resistance to oxidative damage is of particular interest in cancer biology and therapeutics (Klaunig, 2018). The Trx system is overactive in many cancer cells (Selenius et al., 2010; Dong et al., 2016; Roh et al., 2017). The reductive power of the Trx system provides resistance to certain chemotherapies relying on the generation of oxidative stress (Roh et al., 2017). For example, the anti-cancer agent RITA induces oxidative stress in HCT116 colon cancer cells leading to increased TrxR1 tetramer and cross-linked dimer formation (Xu et al., 2015). Newly developed direct inhibitors of TrxR1 have shown efficacy in combination with the cancer chemotherapeutic cisplatin in colon cancer cells (Zhang et al., 2019). Various other small compounds targeting TrxR1 activity are also of interest for cancer treatments (Zhang et al., 2017). Fascinatingly, pre-treatment of the cells with a ROS scavenger and acetate source (Raftos et al., 2007), N-acetyl-l-cysteine, reduced ROS generation, cell death and DNA damage associated with TrxR1 inhibition.

Higher levels of TrxR1 in the serum correlates with shortened overall survival in patients with non-small cell lung cancer (Chen et al., 2017). Proteomic studies of similar cell lines have consistently identified acTrxR1 in HeLa cells, A549 cells and related non-small cell lung cancer cell lines (Choudhary et al., 2009; Weinert et al., 2013; Hornbeck et al., 2015; Wu et al., 2015). Together our data and other studies suggest that TrxR1 acetylation is a potential mechanism to maintain higher Trx system activity. These findings have relevance for the role of TrxR1 acetylation in cancers with poor survival Chen et al. (2017) as well as chemotherapeutic resistance (Iwasawa et al., 2011; Raninga et al., 2016).

## Conclusion

We demonstrated both site-specific and non-specific acetylation of TrxR1 provides robust resistance to oxidative damage even under oxidizing conditions that represent the full range of ROS experienced by TrxR1 in cells. Because TrxR1 plays a vital role in resolving oxidative damage on Trx and other target proteins, TrxR1 and the activity of the Trx system is most important to the cell under conditions of oxidative stress or chemotherapeutic assault. Acetylation of TrxR1 provides a route to increase redox activity, enabling TrxR1 to resist oxidative damage associated with the very reactive oxygen species that the enzyme is tasked to resolve.

## Data Availability

The datasets presented in this study can be found in online repositories. The names of the repository/repositories and accession number(s) can be found at: PRIDE Proteome Xchange, accession number PXD027882.
