# Supplementary material for: Acetylated Thioredoxin Reductase 1 Resists Oxidative Inactivation
Source: Front Chem. 2021 Sep 15;9:747236. doi: 10.3389/fchem.2021.747236 (PMC8479162; doi:10.3389/fchem.2021.747236)
Supplement: Supplementary file 1 [file DataSheet1.docx]

Supplementary Material

# Supplementary Table

**Table S1. MS/MS identified peptides showing lysine acetylation sites in TrxR1.**

| **Residue number** | **Peptide identified** | **-10logP** | **observed m/z** |
| --- | --- | --- | --- |
| TrxR1 + aspirin | | | |
| 28 | ALEGTLSELAAETDLPVVFVK(+42.01)QRK(+21.98) | 67.56 | 1339.7255 |
| 31 | K(+42.01)IGGHGPTLK | 68.67 | 525.3088 |
| 52 | LLK(+42.01)M(+15.99)NGPEDLPK | 82.5 | 706.8766 |
| 88 | YGK(+42.01)KVM(+15.99)VLDFVTPTPLGTR | 63.00 | 727.3947 |
| 176 | K(+42.01)VVY(+42.01)ENAYGQFIGPHR | 68.26 | 654.6655 |
| 176 | K(+42.01)(+43.99)VVYENAYGQFIGPHR | 75.22 | 655.3327 |
| 176 | K(+57.02)(+42.01)VVYENAYGQFIGPHR | 78.52 | 659.6704 |
| 307 | QFVPIK(+42.01)VEQIEAGTPGR | 81.28 | 637.6832 |
| 351 | K(+27.99)(+42.01)IGLETVGVK | 60.77 | 557.3230 |
| 360 | IGLETVGVK(+42.01)INEK | 64.32 | 721.4198 |
| TrxR1 (un-modified) | | | |
| 307 | K(+57.02)(+42.01)VVYENAYGQFIGPHR | 70.47 | 659.6697 |

# Supplementary Figures

**Figure S1. Purified TrxR1 and site-specifically acetylated TrxR1 variants.** Unmodified and acetylated TrxR1 (acTrxR1) variants were purified by nickel column and run on a 15% SDS PAGE, followed by staining with Coomassie. Lanes labels: M indicates a molecular weight marker; wild-type TrxR1 (WT), acTrxR1^K141^ (acK141), acTrxR1^K200^ (acK200), and acTrxR1^K307^ (acK307).

**Figure S2. Oxidation of TrxR1 and site-specifically acetylated TrxR1 variants.** Purified (A) unmodified TrxR1 or (B-D) acetylated TrxR1 variants were incubated with buffer or buffer containing increasing concentrations of H_2_O_2_ ranging from 0 to 500 µM for 1 hour at 37°C. Following incubation, the proteins were visualized by SDS-PAGE followed by Western blotting (anti-TrxR1, above) or Coomassie staining (below). Annotations indicate the molecular weight of TrxR1 monomers and peroxide-induced multimers.

**Figure S3. Quantification of TrxR1 western blot.** The anti-TrxR1 blots (Fig. S2) were used to quantify the level of TrxR1 (A) high molecular multimeric complexes or (B) monomers for wild type (WT) and acTrxR1 variants incubated with 0 to 500 µM H_2_O_2_. Densitometry from the anti-TrxR1 western blot was normalized by the level observed for wild type TrxR1 (A) multimer or (B) monomer. Error bars represent ± 1 standard deviation about the mean of three independent enzyme incubations. Statistical analysis (ANOVA) comparing the amount of protein for each acTrxR1 variant to wild-type TrxR1 at nearly all H_2_O_2_ concentrations demonstrated consistent and significant reductions (by 2 to 5- fold) in high molecular weight complexes (A) for acTrxR1 variants compared to unmodified TrxR1. At 500 µM peroxide, only the acTrxR1^K307^ remains resistant to significant multimerization. Except for a modest and less than 1.5-fold increase in the level of monomeric acTrxR1^K141^ at 0 µM peroxide, the level of monomer observed was not significantly different between wild type and acTrxR1 at each peroxide concentration tested. Significant differences are annotated (* p < 0.05; ** p < 0.005; n.s. – not significant).

**Figure S4. MS/MS spectra of representative TrxR1 peptides showing lysine acetylation following aspirin incubation.** Unmodified TrxR1 was incubated with 15 mM aspirin and then the digested with trypsin and analyzed by LC-MS/MS. These spectra are representative of the complete list of high confidence acetylated peptides identified (Table S1).
